# Chromoblastomycosis Caused by *Phialophora*—Proven Cases from Mexico

**DOI:** 10.3390/jof7020095

**Published:** 2021-01-29

**Authors:** Sarah A. Ahmed, Alexandro Bonifaz, Gloria M. González, Leandro F. Moreno, Nickolas Menezes da Silva, Vania A. Vicente, Ruoyu Li, Sybren de Hoog

**Affiliations:** 1 Center of Expertise in Mycology, Radboud University Medical Center/Canisius Wilhelmina Hospital, 6525GA Nijmegen, The Netherlands; sara3707@gmail.com (S.A.A.); nick.m.silva@gmail.com (N.M.d.S.); 2 Faculty of Medical Laboratory Sciences, University of Khartoum, Khartoum 11115, Sudan; 3 Foundation Atlas of Clinical Fungi, 1214GP Hilversum, The Netherlands; 4 Hospital General de México, “Dr. Eduardo Liceaga”, Dr. Balmis 148, Doctores, 06720 Ciudad de México, Mexico; 5 School of Medicine, Microbiology Department, Universidad Autónoma de Nuevo León, 64460 Monterrey, Mexico; gloria62@hotmail.com; 6 Amsterdam Medical Center, 1105AZ Amsterdam, The Netherlands; leandrofmoreno@yahoo.com.br; 7 Department of Basic Pathology, Federal University of Paraná State, Curitiba 81530-000, Brazil; vaniava63@gmail.com; 8 Graduate Program in Bioprocess Engineering and Biotechnology, Federal University of Paraná, Curitiba 81530-000, Brazil; 9 Department of Dermatology, Peking University First Hospital, Research Center for Medical Mycology, Peking University, Beijing 100034, China; mycolab@126.com or

**Keywords:** chromoblastomycosis, genome, Mexico, molecular identification, *Phialophora americana*, *Phialophora chinensis*, *Phialophora macrospora*, *Phialophora verrucosa*

## Abstract

Chromoblastomycosis is a chronic severely mutilating disease caused by fungi of the order *Chaetothyriales*. Classically, *Phialophora verrucosa* has been listed among these etiologic agents. This species is known to occur in the environment and has been found to cause other infections like phaeohyphomycosis, while reported cases of chromoblastomycosis are scant. *Phialophora* is phylogenetically diverse, and thus retrospective confirmation of etiology is necessary. We studied ten proven cases of chromoblastomycosis from Mexico and further analyzed the population genetics and genomics of the *Phialophora* species to understand their pathogenicity and predilection. The clinical strains were molecularly identified as *Phialophora americana* (*n* = 4), *Phialophora* *chinensis* (*n* = 4), and *Phialophora macrospora* (*n* = 2). No genetic distinction between clinical and environmental strains was possible. Further analysis of strains from diverse origins are needed to address eventual differences in virulence and niche predilection between the species.

## 1. Introduction

Phylogenomic and multilocus approaches have supported the affiliation of agents of the mutilating skin disease chromoblastomycosis to the fungal order *Chaetothyriales*, family *Herpotrichiellaceae* [1]. Members of this family were grouped into six molecularly defined clades, three of which harbor agents of chromoblastomycosis, viz. the bantiana-, carrionii-, and jeanselmei-clades [1]. Recurrent agents of the disease in the literature are classified in the genera *Cladophialophora*, *Fonsecaea*, *Rhinocladiella*, and *Phialophora* [1,2]. All fungi concerned have been implicated in human diseases, but species have their natural habitat in the environment. Upon infection, *Cladophialophora carrionii* and *Fonsecaea* species have unique pathology and exclusively causing chromoblastomycosis, while others are involved in a wide spectrum of clinical conditions, such as mild skin lesions, keratitis, mycetoma, and local or disseminated phaeohyphomycosis [3,4,5].

Chromoblastomycosis is the second most prevalent implantation mycosis worldwide [5]. The disease occurs in dry or humid tropical and subtropical regions around the Tropic of Cancer, with the highest prevalence reported from Australia, Brazil, southern China, India, Madagascar, Dominican Republic, and Mexico [5,6,7]. Chromoblastomycosis is characterized by chronic warty to nodular or cauliflower-like skin lesions and eruptions, with brownish muriform cells (syn. sclerotic cells or Medlar bodies) in the tissue [8,9]. With the distribution of the causative pathogens over several clades, it remains unclear why only these species are able to form muriform cells and cause chromoblastomycosis, while related species in the *Herpotrichiellaceae* mostly provoke erosive infections or are non-pathogenic. *Phialophora verrucosa* is classified in the carrionii-clade, along with *C. carrionii* and its non-pathogenic sibling, *C. yegresii*; the latter species thus far having been isolated from living cactus only [10]. No obvious genomic difference has yet been found to explain pathogenic versus non-pathogenic nature of species in the carrionii-clade, which might indicate that implantation infections by these fungi are coincidental and denote opportunistic nature rather than true pathogenicity [11,12,13]. The same holds true for *P. verrucosa* and relatives: strains have been derived from environmental habitats and from clinical samples, and therefore, it is difficult to distinguish clinically relevant entities from non-virulent members [14,15].

In contrast to its related species, *C. carrionii, P. verrucosa* has been reported to be involved in a wide range of clinical conditions, among which are disseminated and subcutaneous phaeohyphomycosis, eumycetoma, keratitis, and endophthalmitis [10,14,16,17]. Infections attributed to *P. verrucosa* have mostly been reported from apparently healthy individuals from subtropical climate zones including China, Japan, Colombia, Brazil, Mexico, U.S.A., and Libya [8,14,18,19,20]. Occasionally, patients have immunological disorders such as CARD9 mutation [21,22], impairing differentiation of Th_17_ immune cells and leading to extended, erosive case of phaeohyphomycosis [22].

*Phialophora verrucosa* is the type species of the genus *Phialophora* and was described from a patient with skin lesions and muriform cells in tissue in the USA [8,22]. *Phialophora americana* was later described as an anamorph of *Capronia semiimmersa* and found to be a sister to *P. verrucose* [23]. Li et al. [14] noted further diversity and defined seven molecular siblings. Besides *P. verrucosa* and *P. americana*, *P. chinensis*, *P. ellipsoidea*, *P. expanda*, *P. macrospora*, and *P. tarda* were introduced. The term ‘*Phialophora verrucosa* complex’ is available for *P. verrucosa* and allied species [24]. Interestingly, out of 118 strains phenotypically recognized as *P. verrucosa*, only six were found by Li et al. [14] to belong to *P. verrucosa* sensu stricto, and none of these originated from chromoblastomycosis. Furthermore, several morphologically similar taxa (*P. reptans*, *P. oxyspora*, *P. europaea*, *P. ambigua*) were excluded as phylogenetically distant from the genus *Phialophora* [25].

Proof of etiology of chromoblastomycosis by *P. verrucosa* according to present standards is lacking. Therefore, we collected *P. verrucosa*-like strains from proven cases of chromoblastomycosis from Mexico, in order to properly identify those strains according to the species concept of Li et al. [14] and to study population structures within and across related species. Comparative genomics of the *P. verrucosa* complex and related species in the carrionii-clade (*C. carrionii*, *C. yegresii*) was performed to determine genetic signatures and potential virulence factors shared by these fungi.

## 2. Materials and Methods

### 2.1. Ethics Statement

This study was reviewed by the Review Board and Ethics Committee of the Hospital General of México. Due to the retrospective nature of the study, the review board waived the requirement for informed consent. Patients’ data were kept anonymous to ensure confidentiality and privacy.

### 2.2. Patients and Strains

Ten phenotypically identified *P. verrucosa* strains recovered from patients with chromoblastomycosis in Mexico were analyzed. Demographic and clinical data were summarized in Table S1. Case studies for the identified species were reported; a single case was selected randomly per species. Isolates were grown on Sabouraud’s Glucose Agar (SGA, Oxoid, Basingstoke, Hampshire, England) and maintained at 30 °C. Micromorphology and growth characteristics were studied on 2% Malt Extract Agar (MEA, Oxoid), Oatmeal Agar (OA, 2% oat meal), and Potato Dextrose Agar (PDA, Oxoid) plates incubated for 3 weeks at 25 °C. Slides were examined with a Nikon Eclipse 80i (Nikon, Tokyo, Japan) microscope equipped with a Nikon digital sight DS-5M camera, and micrographs were captured with NIS-Elements software.

For DNA extraction, a cetyltrimethyl ammonium bromide (CTAB) with glass beads method was applied according to Möller et al. [26]. The ITS region was amplified and sequenced with primers, ITS1 and ITS4 [27]. A fragment of the β-tubulin (*TUB2*) gene was amplified and sequenced with primers, Bt2a and Bt2b [28]. The ITS and *TUB2* sequences were aligned separately with 39 *Phialophora* reference sequences retrieved from GenBank, mostly from Li et al. [14], and then combined in a single dataset using the Dataconvert software. Phylogenetic position of the clinical strains was determined using Maximum Likelihood analysis in RAxML v8.2.10 [29]. The GTR (General Time Reversible) model of nucleotide substitution and 1000 ML bootstrapped pseudoreplicates were applied. Resulting tree was rooted with *C. carrionii* strains, CBS 160.54 and CBS 117906, and edited in MEGA v6.0 [30].

### 2.3. Population Genetics

Concatenated ITS and *TUB2* sequences of 139 strains were used to study the population genetics of *Phialophora*. The number of polymorphic sites (S), haplotype diversity (Hd), and nucleotide diversity (π) were calculated in DNASP v5.10, while gaps and missing data were excluded. The haplotype network was constructed in Network v10 (Fluxus-Technology, Colchester, Essex, England) using Median-joining method. Evidence of recombination was investigated by performing pairwise homoplasy index test (Phi) and Neighbor-Net method implemented in Splitstree v4.8 [31].

### 2.4. Genome Analysis

We compared the protein set of the neotype strain of *P. verrucosa* (MSED00000000) with *P. americana* (JYCC01000000), *C. carrionii* (AOFF01000000), *C. yegresii* (AMGW01000000), *Fonsecaea pedrosoi* (JYBS00000000), and *F. monophora* (LVKK00000000). The genomes and their respective protein sequences were downloaded from the National Center for Biotechnology Information (NCBI) database. Ortholog clusters were identified with OrthoVenn and default parameters (E-value 1 × 10^−5^, Inflation value 1.5) [32]. The gene ontology (GO) categories were also determined using OrthoVenn, and the hypergeometric test with a *p*-value < 0.05 was applied to find enriched GO in the clusters.

## 3. Results

### 3.1. Case Reports

#### 3.1.1. Case Presentation of *P. americana* (Isolate dH 24521)

**Figure 1:**
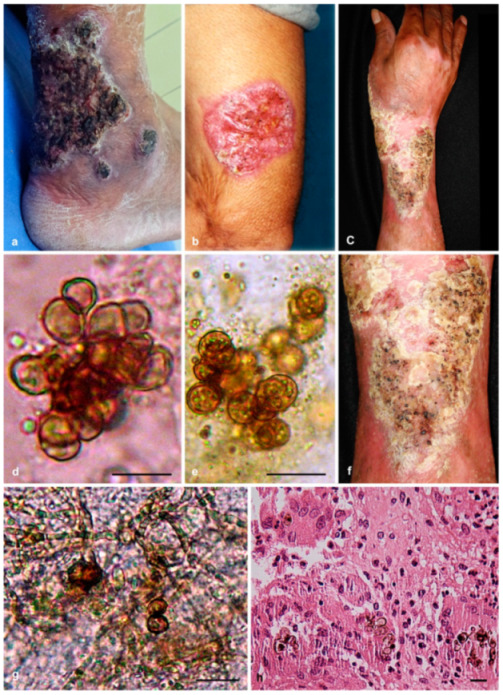
Clinical presentation, direct examination, and histology of *Phialophora* cases. (**a**,**d**) The case of *Phialophora americana*; (**b**,**e**) The case of *Phialophora chinensis*; (**c**,**f**–**h**) The case of *Phialophora macrospora*. Scale bar = 10 µm.

A 42-year-old male patient originating from Juchitán Oaxaca (730 km southeast of Mexico City) was a farmer by occupation dedicated to planting corn and chili-pepper. He presented with localized dermatosis of the lower left limb affecting the heel region, consisting of a verrucous-nodular plaque of about 15 × 10 cm and two nodular lesions, all with erythematous borders. The squamous lesions were dark in appearance with black dots in the perimeter. The patient reported moderate pruritus and the lesion’s evolution over 1.5 years after trauma with a tillage instrument. On direct examination, multiple muriform cells and brown hyphae surrounded by a tuberculoid granuloma were observed. The culture was phenotypically identified as *P. verrucosa* (Figure 1, Table S1).

#### 3.1.2. Case Presentation of *P. chinensis* (Isolate dH 24531)

A 53-year-old male farmer from Orizatlán, Hidalgo, Mexico, presented with a painless lesion on the left arm that had developed over 2 years. The lesion was an erythematous plaque with well-defined margins and covered by black dots. The lesion had started as a small papule following traumatic inoculation of a plant while working. No satellite lesions were observed. At physical examination, blood pressure, pulse, and temperature were within normal limits. The patient was otherwise healthy with no underlying disease, which might have led to impaired immunity. Several ointments had been used to treat the condition, with no improvement.

Direct potassium hydroxide (20%) wet mounts of the lesion revealed brown, thick-walled, septate muriform cells. Histopathology with hematoxylin and eosin staining of the biopsy tissue showed hyperkeratosis and granulomatous response with histiocytes, polymorphonuclear cells, and muriform cells. These results confirmed the clinical diagnosis of chromoblastomycosis. The isolated fungus was phenotypically identified as *P. verrucosa* (Figure 1, Table S1).

#### 3.1.3. Case Presentation of *P. macrospora* (Isolate dH 24520)

A 48-year-old male farmer, originating from and residing in Papantla Veracruz (300 km northeast of Mexico City), dedicated to growing corn and sugar cane, presented with localized dermatosis of the right upper limb, affecting the forearm. The lesion consisted of a nodular-verrucous plaque measuring approximately 18 × 8 cm with erythematous borders, formed by squamous lesions with black dots and in a central area of hypopigmentation. The patient reported moderate pruritus during 2-year evolution. Upon direct histopathological examination, multiple muriform cells and brown hyphae in a suppurative granuloma were observed. The culture was identified phenotypically as *P. verrucosa* (Figure 1, Table S1).

### 3.2. Morphology and Phylogeny

**Figure 2:**
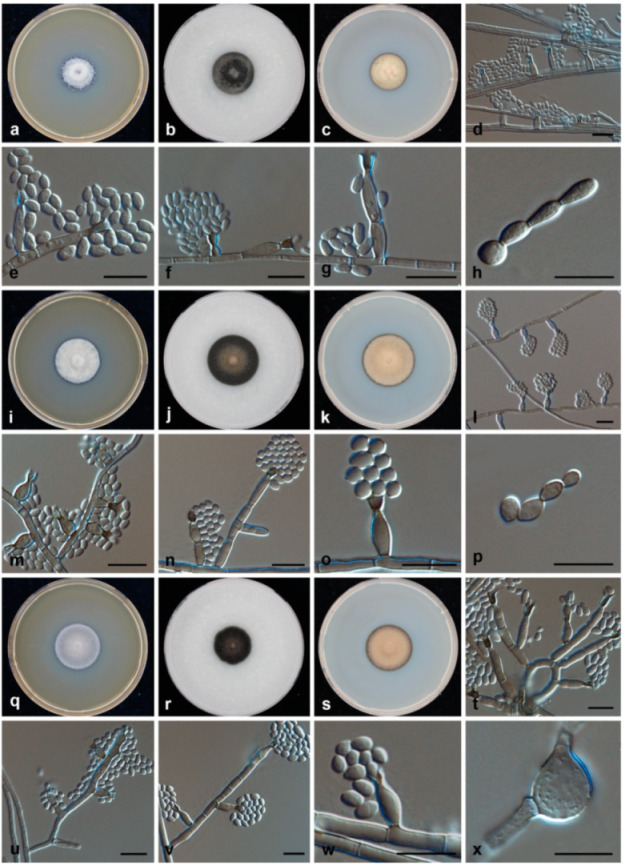
Growth and micromorphology of the clinical isolates. (**a**–**h**) *Phialophora americana*; (**i**–**p**) *Phialophora chinensis*; (**q**–**x**) *Phialophora macrospora*. Scale bars = 10 μm.

After three weeks of incubation, the ten clinical strains grew as olivaceous grey to black colonies, with variable growth rates. Microscopically, all strains showed similar morphological features in that hyphae were brown, septate, and branched; phialides were lateral or terminal, flask-shaped or elongated to cylindrical, with characteristic vase- to funnel-shaped darkly pigmented collarettes; conidia were hyaline, smooth-walled, and spherical to ellipsoidal (Figure 2).

**Figure 3:**
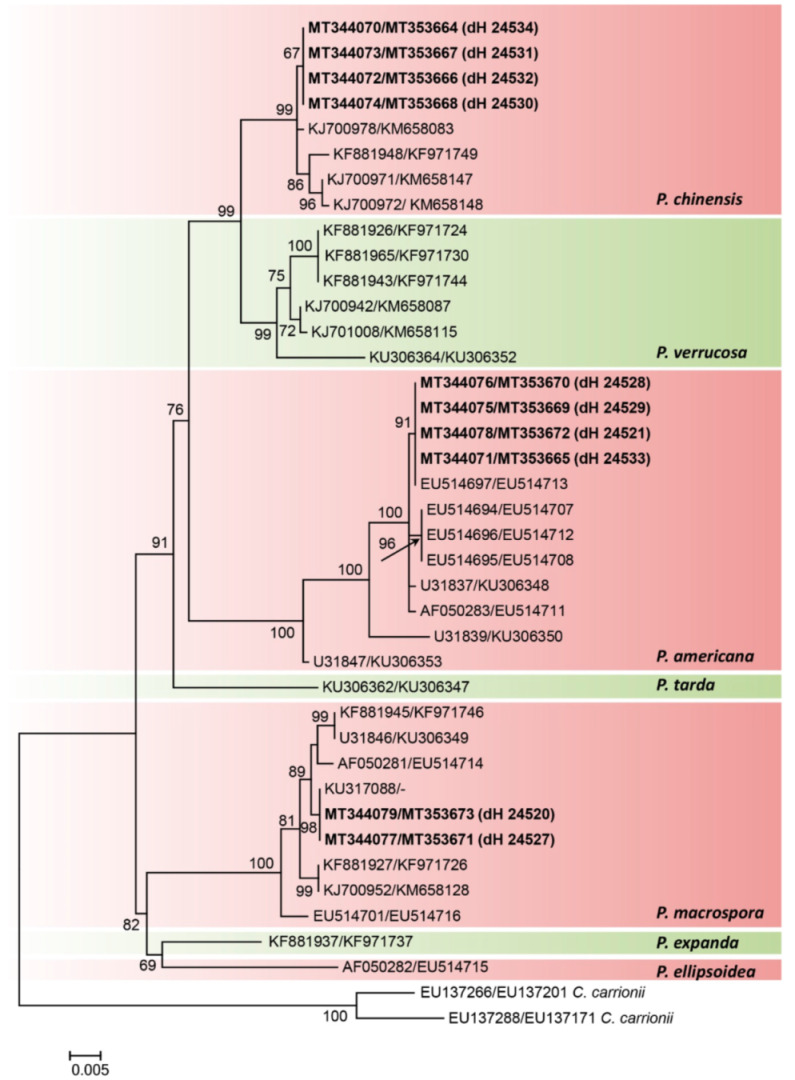
Combined Internal Transcribed Spacer (ITS) and β-tubulin (*TUB2*) phylogeny of the genus *Phialophora* obtained by maximum likelihood method (bootstrap values are indicated at the nodes). Sequences of the clinical isolates recovered from the cases presented in this study are indicated in bold. *Cladophialophora carrionii* is used as an outgroup. Taxa labels are the GenBank accession numbers of ITS/*TUB2*.

To determine the species identity of the clinical strains, a phylogenetic approach was applied. In a two-gene tree, the seven *Phialophora* species as defined by Li et al. [14] were monophyletic and clearly separated (Figure 3). None of our clinical strains clustered with *P. verrucosa* s. str.; four of them were found to belong to *P. americana* and four to *P. chinensis*, while two matched with *P. macrospora*. Within these species, our clinical strains formed distinct intra-specific subclades. Our *P. macrospora* strains clustered (98% bootstrap support) with a strain from a chromoblastomycosis patient from Brazil dH 12667 (KU317088, Figure 3). The *P. americana* strains from our patients clustered at 91% with strain UAMH 10876 (EU514697), which was originally identified as *Capronia semiimmersa* and was isolated from wood in the U.S.A. The *P. chinensis* strains clustered at 99% with several strains from China, and most of them were derived from the environment.

### 3.3. Population Genetics

**Figure 4:**
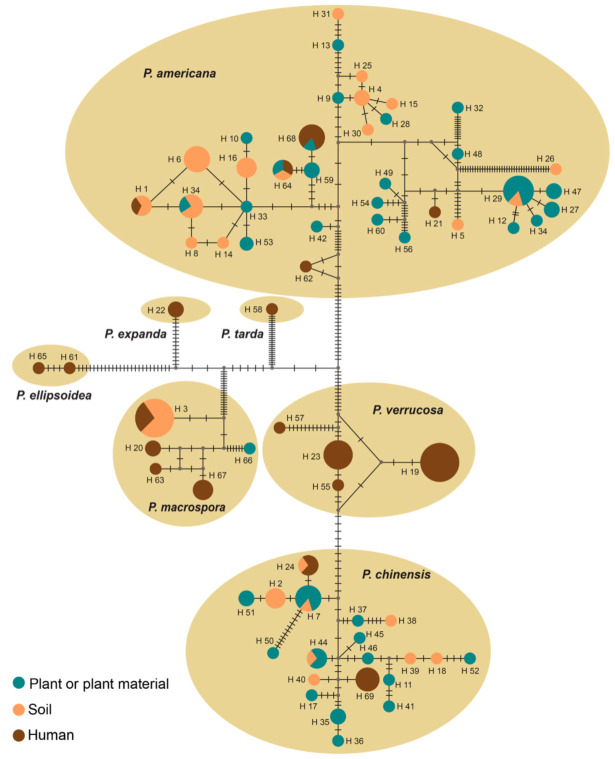
Haplotype network of *Phialophora* species resulting from concatenated ITS and *TUB2* sequences of 139 strains. Each pie chart represents a unique haplotype and the size is proportional to the frequency of the haplotype. Small grey dots show the median vectors (mv), which represent internal haplotypes that were not present in the dataset. Dashes in the lines connecting the haplotypes represent mutation steps.

Genetic diversity in *Phialophora* was assessed using the concatenated ITS and *TUB2* genes of 139 strains with total alignment length of 997 bp. In the dataset, 158 sites were variable, of which 48 were singleton and 137 were parsimonious informative. The haplotype network divided the isolates into 69 haplotypes (Hd = 0.9784, π = 0.03819; Figure 4). *Phialophora ellipsoidea*, *P. expanda*, and *P. tarda* were presented with only a single or with two strains, and were grouped into four 1–2-species haplotypes. Among the species represented by >2 strains, *P. verrucosa* (*n* = 19) showed the lowest diversity; strains were grouped into four haplotypes (Hd = 0.591, π = 0.00508). All *P. verrucosa* strains were from clinical origin causing phaeohyphomycosis. Low diversity was also noted in *P. macrospora* (*n* = 17); the isolates were divided into five haplotypes (Hd = 0.640, π = 0.00465). In contrast, the population of *P. chinensis* (*n* = 35) showed high diversity with 20 haplotypes (Hd = 0.955, π = 0.00638). The clinical strains of *P. chinensis* were grouped into two haplotypes; Hap 69 contained our strains from Mexico and Hap 24 contained two strains from China, one clinical and one environmental. The highest diversity was revealed in the *P. americana* (*n* = 63) population, with 36 haplotypes (Hd = 0.973, π = 0.01195) consisting of both clinical and environmental isolates. The environmental strains mostly originated from China, whereas the clinical strains were from North and South America and grouped into five haplotypes. *Phialophora americana* Hap 64 was the only haplotype in the complex that comprised clinical, plant, and soil isolates. Clinical and plant-associated isolates originated from the U.S.A., while the one from soil was isolated in Brazil. Although our clinical strains in *P. americana* and *P. chinensis* grouped into distinct haplotypes, presence of Hap 64, 68, and 24 among the population indicated equal pathogenic potential of members of the two species. Thus, the distinction of species in *Phialophora* into clinical and environmental categories seems invalid.

**Figure 5:**
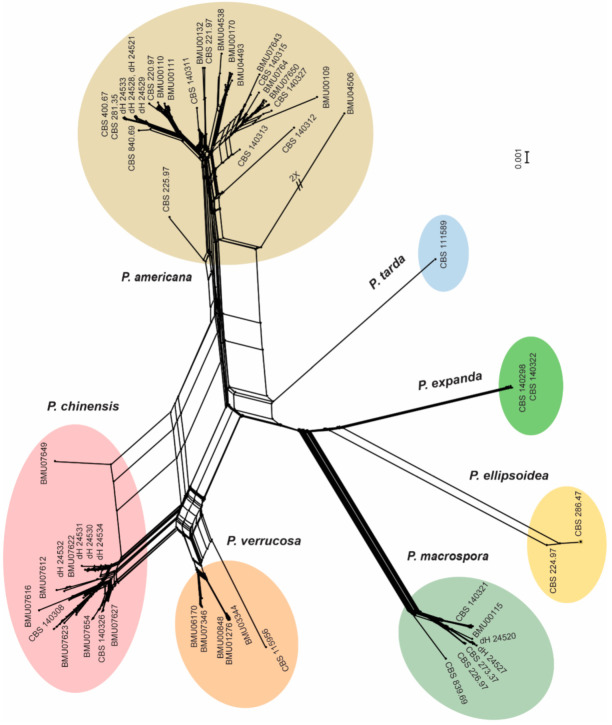
Neighbor-Net split decomposition network of *Phialophora* species based on ITS and *TUB2* sequences of 139 strains. The splits between branches in the network indicate the locations of incongruence and the possibility of recombination. Some of the names of taxa were removed to enhance the clarity of the graph.

Combined ITS and *TUB2* sequences were also used to study interspecies recombination among *Phialophora* populations. In the Neighbor-Net (Figure 5), members of each species clustered together, but the diversity was clearly visible in the *P. americana* and *P. chinensis* populations. The network also showed evidence of interspecies recombination in *P. americana* and *P. chinensis*, which is indicated by formation of parallelograms. With Phi test, a similar result was obtained with statistically significant evidence for recombination in *P. americana* (*p* = 5.168 × 10^−7^) and *P. chinensis* (*p* = 1.4511 × 10^−4^). Notably, evidence of recombination was limited to species that preponderantly contained environmental isolates. In the other species with a bias to clinical isolates, no evidence of recombination was found (*P. verrucosa*, *p* = 1.163; *P. macrospora, p* = 0.29649).

### 3.4. Comparative Genomics

**Figure 6:**
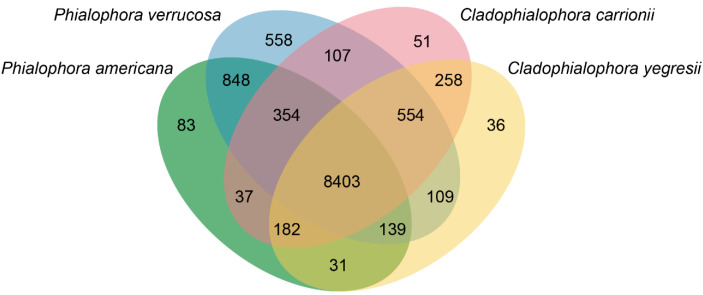
Whole-genome comparison of *Phialophora verrucosa* and *P. americana* with *Cladophialophora carrionii* and *C. yegresii*; all species are members of the carrionii-clade in *Chaetothyriales*. *C. yegresii* represents a non-clinical species, while *P. verrucosa* and *C. carrionii* are clinical. Numbers in the Venn diagram are the unique and shared genes among the species.

Comparison of *P. verrucosa* (genome size of 35.46 Mb) and *P. americana* (31.62 Mb) with neighboring species, *C. carrionii* (29.99 Mb) and *C. yegresii* (27.9 Mb), in the carrionii-clade revealed a total of 8403 ortholog clusters shared among the four species. *Phialophora verrucosa* possesses the highest number of specific ortholog clusters (558) followed by *P. americana* (38), while *C. carrionii* and *C. yegresii* have only 51 and 36 specific clusters, respectively (Figure 6). Functional annotation and posterior statistical analysis of the orthologs exclusively found in *P. verrucosa* revealed that these clusters are enriched by proteins involved in the galactose metabolic process (GO:0006012) and transcription regulator activity (GO:0006351).

In order to further investigate genes potentially engaged in pathogenicity that were shared between different species of *Herpotrichiellaceae*, we rebuilt the ortholog clusters by including the proteins of *F. pedrosoi* and *F. monophora*, two agents of chromoblastomycosis. *Fonsecaea pedrosoi* causes chomoblastomycosis exclusively, while *F. monophora* has additionally been reported from cerebral phaeohyphomycosis. Using this new dataset, the number of shared orthologs between the analyzed species was 7609 and only few clusters were found specific for every species (Figure S1). Remarkably, no species-specific cluster of orthologs were found in *C. carrionii* and only two were found in *C. yegresii*, while 50 were found specifically for *P. verrucosa* and 59 for *P. americana*. Clusters that were shared among the pathogenic species totaled 200 and mainly included proteins that were involved in biological processes. The molecular functions of the shared orthologs include proteins involved in oxidoreductase, transferase, hydrolase, peptidase, and monooxygenase activity, in addition to transporter, antioxidant, and ion binding activity. Although some of these proteins might contribute to the pathogenicity of chromoblastomycosis agents, the exact virulence determinants in these agents remain enigmatic. On the other hand, among the orthologs specific to *P. verrucosa*, we found a cluster (GO: 0009405) of cytochrome P450 monooxygenase that has been linked to niche adaptation and pathogenicity.

## 4. Discussion

The fungal genus *Phialophora* dates back to 1915, with *P. verrucosa* as the type species, but molecular characterization to define and diagnose species borderlines in a modern sense was performed only recently [8,14,22]. The newly defined species are molecular siblings as they show reproducible genetic differences, but are phenotypically similar except in their growth rates and some minor differences in the shape of collarettes [14]. We found that these differences are strain-dependent; no distinct phenotypic feature was recognized among the clinical strains in our study. Li et al. [14] reported possible differences in predilection or habitat-specificity among *Phialophora* species; *P. americana* and *P. chinensis* were found to be preponderantly environmental, while *P. verrucosa* and other rare species tended to be of clinical origin. In our data, *P. verrucosa* is still exclusively clinical, but most of our Mexican clinical strains (*n* = 10) cluster in environmental species: four in *P. chinensis*, and four in *P. americana*, while two belong to the clinical species *P. macrospora*. Thus, agents of chromoblastomycosis can also be found in environmental species, and ecological distinction proves to be of limited diagnostic value. Therefore, reference to *P. verrucosa* as a species complex in the sense of Chen and coworkers is warranted [33]. Possibly, data of Li et al. [14] were affected by sampling bias, since most of the isolates in their study were from Asia. Nevertheless, *P. verrucosa* s.str. still appears to be prevalently clinical, but does not contain any proven agent of chromoblastomycosis. In contrast, *P. chinensis* is prevalently environmental [14], but does provoke chromoblastomycosis occasionally.

In general, environmental isolation of opportunistic black yeasts in *Chaetothyriales* is known to be of great difficulty [34]. This differs between species; for example, genomic data of *Rhinocladiella mackenziei* indicate environmental extremophily but, as yet, it has only been isolated from human brain [35]. This might also explain the absence of environmental recovery of *P. verrucosa,* despite extensive environmental sampling [14]. In older literature, authors reported successful isolation of *P. verrucosa* using a mammal vector as enrichment method, but the identity of their isolates has not been confirmed by molecular methods [15].

*Phialophora verrucosa* is not known to have sexuality, whereas *P. americana* produces a sexual morph characterized by small, dark, setose ascomata and septate ascospores [2]. In line with these data, *P. americana* was confirmed to have both MAT1-1 and MAT1-2 genes in a single genome [36], and we found evidence of recombination using both Phi test and Neighbor-Net approach; in addition, high haplotype diversity was noted. The population structure of the *P. verrucosa* complex is reminiscent of that of the *Sporothrix* species, the cause of another mutilating fungal skin disease, sporotrichosis [37]. While *S. schenckii* showed a mixed origin with high diversity, *S. globosa* and *S. brasiliensis* resembled clonal offshoots [37,38]. The low diversity in the latter species has been linked to recent divergence from the ancestral species, *S. schenckii* [37]. In analogy, *P. americana* seems to be ancestral to *P. verrucosa*, which may have a recently acquired an enhanced ability to infect humans. Notably, several patients with CARD9 mutations suffered from an aggressive infection of the rare species *P. verrucosa* s.str. [21].

At the present, whole-genome sequences of *P. verrucosa* s.str. and other species in the carrionii-clade have become available. When comparing the genomes of *P. verrucosa* with chromoblastomycosis-causing species, *C. carrionii*, *F. pedrosoi*, *F. monophora*, and the environmental species *C. yegresii*, we could not accurately determine which proteins are engaged in pathogenicity [36], as it proved to be difficult also in other comparative genomic studies involving black yeasts [13,36]. Furthermore, by comparing the number of unique proteins in every species, it was obvious that *C. carrionii* (chromoblastomycosis) shared a higher number of proteins with *C. yegresii* (environmental) than with *P. americana* and others, suggesting that these differences were more phylogenetic than reflecting similarities in ecology. Although the prime ecological niches of species in the *P. verrucosa* complex remains unknown, the high number of species-specific ortholog clusters found in *P. verrucosa* and *P. americana*, including for e.g., genes functionally related to membrane transport of small solutes and CYP450s, could be responsible for the adaptation to a distinct functional niche and consequently have determined evolution of separate lineages. Transporters and CYP450 have been suggested to play a crucial role for the adaptation of black yeasts to extreme environments [36].

The current recommendation for treating black yeast cutaneous infections, including chromoblastomycosis and phaeohyphomycosis, is itraconazole (ITZ, 400 mg) alone or in combination with conventional surgery or cryosurgery [39]. Therefore, all our patients were treated with ITZ and showed clinical improvement during follow up, despite slightly elevated ITZ minimum inhibitory concentration (MIC) of the infecting isolates. The highest MIC to ITZ was a strain of *P. chinensis* (2 µg/mL), and accordingly, 250 mg of terbinafine was given. The patient improved after two months and was cured after one year. In a study of 46 strains of *Phialophora* performed by Li et al. [40], 50% of the strains were inhibited by 0.25 µg/mL of voriconazole and 0.125 µg/mL of posaconazole, whereas for amphotericin B (AMB), the MIC_50_ was 4 µg/mL. These AMB data are not in agreement with our test results. The highest MIC among the four strains tested was 0.5 µg/mL. This variability requires further evaluation by testing larger numbers of well-characterized strains from diverse origins.

## Data Availability

The data presented in this study are available in “Chromoblastomycosis Caused by Phialophora—Proven Cases from Mexico.

## Supplementary Materials

The following are available online at https://www.mdpi.com/2309-608X/7/2/95/s1. Figure S1: Venn diagrams showing unique and shared orthologous clusters between genomes of *P. verrucosa*, *P. americana*, *C. carrionii*, *C. yegresii*, *F. pedrosoi*, and *F. monophora*. Table S1: Clinical data and strains.
