# Supplementary material for: Chromoblastomycosis Caused by Phialophora—Proven Cases from Mexico
Source: J Fungi (Basel). 2021 Jan 29;7(2):95. doi: 10.3390/jof7020095 (PMC7910919; doi:10.3390/jof7020095)
Supplement: Supplementary file 1 [file jof-07-00095-s001.pdf]

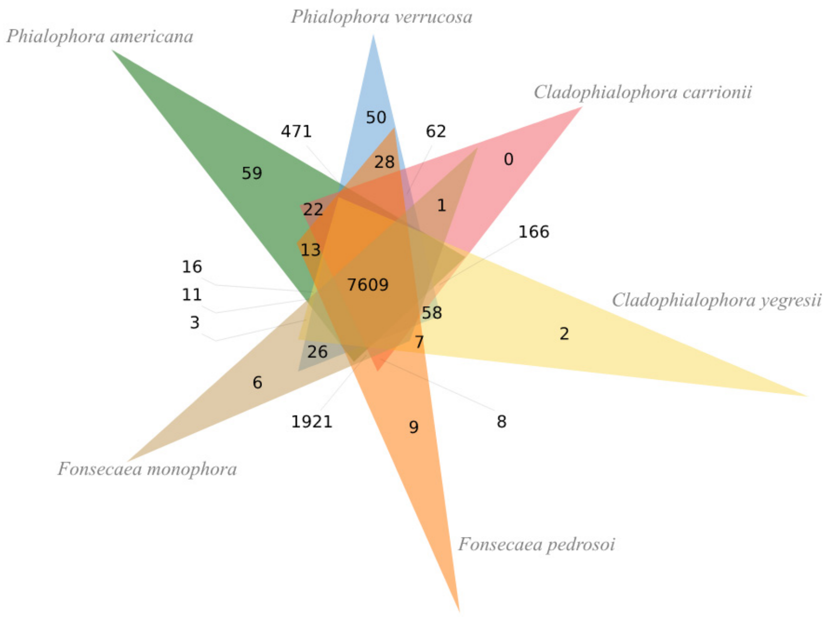

Figure S1. Venn diagrams showing unique and shared orthologous clusters between genomes of *P. verrucosa*, *P. americana*, *C. carrionii*, *C. yegresii*, *F. pedrosoi*, and *F. monophora*.

Table S1. Clinical data and strains *.

Strain	Identification	M/F	Age	Site	Evolution (y)	Origin	Clinical form	Treatment duration	Antifungal susceptibility (μg/ml), treatment, and outcome
dH 24520	*P. macrospora*	M	48	Right upper limb	2	Papantla, Veracruz	Nodular & verrucous	1 y + 6 mo	MICs: Not done. Treatment: ITC 400 mg/day + cryosurgery; clinical & mycological cure.
dH 24521	*P. americana*	M	42	Lower left limb	1	Juchitán, Oaxaca	Nodular & verrucous	1 y + 6 mo	MICs: Not done. Treatment: ITC 300 mg / day + cryosurgery; clinical & mycological cure.
dH 24527	*P. macrospora*	M	41	Lower right limb	2	Huejutla, Hidalgo	Nodular & verrucous	1 y + 2 mo	MICs: Not done. Treatment: ITC 400 mg / day; clinical improvement.
dH 24528	*P. americana*	M	56	Left arm	4	Tamazunchale, San Luis Potosí	Nodular & verrucous	1 y + 2 mo	MICs: Not done. Treatment: ITC 400 mg / day; clinical improvement and still under treatment.
dH 24529	*P. americana*	M	42	Lower right limb	1	General Zaragoza, Nuevo León	Nodular & verrucous	1 y + 4 mo	MICs: Not done. Treatment: ITC 400 mg / day, cryosurgery; clinical & mycological cure.
dH 24530	*P. chinensis*	M	39	Right arm	2	Gomez Farías, Tamaulipas	Nodular & verrucous	3 mo	MICs: 0.25 AMB, 0.5 VRC, 0.5 PSC, 1 ITC Treatment: ITC 400 mg/day, follow up was lost at 3 months.
dH 24531	*P. chinensis*	M	53	Left arm	2	Orizatlán, Hidalgo	Nodular & verrucous	1 y	MICs: 0.25 AMB, 0.25 VRC, 0.25 PSC, 0.5 ITC. Treatment: ITC 400 mg/day; clinically improved after 4 months, after 1 year clinical & mycological cure.
dH 24532	*P. chinensis*	M	47	Right arm	3	Axtla de Terrazas, San Luis Potosí	Nodular & verrucous	1 y	MICs: 0.5 AMB, 0.5 VRC, 1 PSC, 2 ITC. Treatment: ITC 400 mg/day + terbinafine 250 mg/day; clinically improved after 2 months, after 1 year clinical and mycological cure.
dH 24533	*P. americana*	M	52	Right leg	4	Tampamolón Corono, San Luis Potosí	Nodular & verrucous	3 mo	MICs: 0.25 AMB, 0.5 VRC, 0.5 PSC, 1 ITC. ITC 400 mg/day, follow up was lost at 3 months; little improvement
dH 24534	*P. chinensis*	M	74	NA	7	Xichu, Guanajuato	Tumoral & cystic	NA	NA

* (M/F), M=Male, F=female; Amphotericin B (AmB), voriconazole (VRC), posaconazole (PSC), and itraconazole (ITC); NA, data not available.
